# Drowning rates among children and adolescents (aged 7–17) in Israel during the years 2008–2018

**DOI:** 10.1186/s12889-023-16671-y

**Published:** 2023-09-02

**Authors:** Sigalit Abihasira, Daniel S. Moran, Daniela Orr, Uri Eliyahu

**Affiliations:** 1 Department of Health Systems Management, Faculty of Health Sciences, Ariel University, 40700 Ariel, Israel; 2 Beterem – Safe Kids Israel organization, 30 Hasivim St., Kiryat Matalon, P.O.B. 7050, 4917001 Petah-Tikva, Israel; 3 Department of Nutrition, Faculty of Health Sciences, Ariel University, 40700 Ariel, Israel

**Keywords:** Safety, Swimming environment, Designated bathing sites, Minorities, Awareness

## Abstract

**Objective:**

Drowning is one of the leading causes of death among children and youth worldwide. This study aims to examine differences in the rates of drowning (fatal and non-fatal drowning) among children and youth in Israel stratified by age, sex, sector, place of drowning, and the drowning outcome. In addition, we compared the results of studies reported in other countries in specific age groups based on statistics of about 100,000 drowning cases.

**Method:**

A statistical analysis of 474 drownings between 2008 and 2018 was conducted. All cases refer to youngsters aged 7–17 in the State of Israel. Statistical analysis was performed on data obtained from the Beterem – Safe Kids Israel organization and from the Israel Central Bureau of Statistics. Disparities between groups within the examined population were analyzed based on gender, sector (Jewish versus non-Jewish), and drowning site.

**Results:**

Of the 474 drownings that occurred during 2008–2018, 38.4% ended in death. 79% of the cases occurred in pools. The Arab minority sector (21.1% of the general population) accounted for 25.1% of all drownings, males accounted for 70.5% of the drowning cases, and the age group with the most drownings (48.5%) was that of 15–17 years. The Jewish population was involved in more than 75% of drownings in places designated for bathing and in more than 83% of all disaster scenarios, whereas the Arab minority was involved in more than 61% of drownings in places not designated for bathing.

**Conclusions:**

The results are comparable to those of other studies worldwide. Boys drown twice as much as girls, mainly in the age group of 15–17. This may be explained by overconfidence in boys and a tendency to overestimate their actual swimming abilities. Most drownings occur in pools. Drowning among the Jewish population occurs mainly in designated bathing sites.

**Practical applications:**

The findings can and, in fact, must be used to inform and educate the younger generation as to the potential dangers involving bathing in designated bathing sites.

## Introduction

Drowning poses a severe threat to public health, as it is one of the leading causes of death among children and youth around the world [1]. It is caused mainly by unsafe behavior in the water, lack of supervision, and location.

The last decade has seen an increase in the number of drowning cases in children and adolescents. In the State of Israel, drowning is the second leading cause of death among children and adolescents (aged 0–17) [2].

Drowning is among the ten main causes of death for children and young people aged 1–24 worldwide. It is the sixth most common cause of death in the world among children aged 5–14 years. Over 90% of drowning deaths occur in low- and middle-income countries. In the year 2019, nearly 236,000 people lost their lives by drowning [1].

According to the 2017 WHO report [3], in the Middle East, fatal drowning of children and adolescents is the fifth and sixth cause of death. In Southeast Asia, it is the second to third leading cause of death. In various countries in Europe, fatal drownings in this age group are the fourth and fifth causes of death. In the African continent, drowning is the ninth to the tenth cause of death among children and adolescents (aged 5–15), and it is the leading cause of mortality in the Western Pacific region, while in the Americas, drowning is the third leading cause of death.

A variety of studies proposed in the literature describe the rate of fatal and non-fatal drowning; for example, a study in the United States that examined hospitalization due to drowning among children and adolescents (aged 0–19) found that drowning rates for both genders decreased from 4.7 in 1993 to 2.4 in 2008, a reduction of 49%. This trend was also found in fatal drowning, exhibiting a reduction from a rate of 0.5 between 1993–1994 to 0.3 between 2007–2008 [4].

In the United States in 2016, out of 20,360 child and adolescent (aged 1–19) deaths, approximately 4.9% occurred due to drowning, i.e., an incidence rate of 1.27 [5]. Fatal drowning and drowning risk factors from 15 countries in Africa between the years 1982–2017 show that the most prominent drowning risk factors among children and adolescents are younger age, gender (males; double chance), ethnicity, alcohol consumption, unsafe access to water bodies, age, and carrying capacity of the boat, weather, and the summer season [6]. A high population drowning rate among minorities can be attributed to differences in age, gender, drowning location, access, supervision, swimming, and communication skills (Martin, N.T., 2010).

Non-fatal drowning rates among U.S. minority groups, young people under 21 years of age at drowning sites (swimming pools, natural reservoirs, and other sources), age groups, gender, and population segments (Caucasian vs. African-American and Hispanic population), the average age for drowning was 10.9. In pools, the average age was 9.6; in natural water reservoirs—14.4; and in other sources, it was 10.2. About 78% or more were males. The African American minority population made up about 30% of all drownings, Hispanics about 45%, the Caucasian population a little over 10%, and other groups made up approximately 15%. In the age group of 5–9, the total drowning rate was 9.0, while among Caucasians, the rate was 3.8, and among African Americans and Hispanics, over 15.0. In the age group of 10–14, the overall drowning rate was 7.9. Among Caucasians, the rate was 3.4, while among minorities, the rate was above 13.0. In the age group of 15–19, the overall drowning rate reached 7.4. 3.1 occurred among the Caucasian population, whereas among minorities, the rate was more considerable, reaching 12.0 [7].

However, not all drowning events are fatal. For example, an Australian report published in 2017 summing up 13 years of tracking non-fatal drowning events concluded that the non-fatal drowning rate was 2.71 times higher than that of fatal drownings. In the age group of 5–14, the rate was over six times higher, while in the age group of 15–17, the rate reached 2.89 times higher [8].

In this study, the sources of information include data on death rates from drowning and drowning in general among children and teenagers. The study examines disparities in fatal and non-fatal drowning rates in Israel among children and youth (7–17 years old) during the years 2008–2018 while stratifying by background factors, i.e., gender, sector, and drowning sites. In addition, the results of the study are compared to the results of other studies in the world in order to examine where Israel is in relation to other countries in the rate of fatal and non-fatal drowning.

The study is the first and pioneering study in the State of Israel, attempting to find differences between the different groups and an explanation for those differences; for example, differences between the Jewish and Arab sectors may provide an indication of cultural differences between the populations and a connection to the drowning. We also aim to explore whether the fatal and non-fatal drowning outcomes for the adult age group (15–17) are a result of risky behavior or other reasons.

## Materials and methods

### Study population and design

This study is a retrospective descriptive study of fatal and non-fatal drowning data among a population of children and adolescents between the ages of 7–17 during the years 2008–2018. The drowning cases were analyzed based on demographic data and included 474 cases of fatal (105) and non-fatal (369) drowning at bathing sites or various bodies of water in the State of Israel.

### Data collection

Drowning cases of children and teenagers aged 7–17 between 2008 and 2018 were analyzed based on demographic data. The analysis was carried out using the data from the association "Beterem—Safe Kids Israel" on the number of drowning cases in the years 2008–2018. The "Beterem" dataset includes data from three sources. The first is a database that includes information related to drowning from the emergency departments of ten hospitals. The second is a compilation of pediatric imprinting data from eleven other hospitals. The 21 hospitals reported in the data make up 70% of all public and private hospitals in Israel. The third source is a database documenting injuries reported in the written and online media. The data collected from the hospitals include the ages 0–17 years. In this study, the selected age range is 7–17, similar to the age groups reported in the literature for drowning rate comparison. Population size data were obtained from the Central Bureau of Statistics database [9]. These data were used to estimate the rate of drowning incidents during 2008–2018 in the various age groups and corrected to value per 100,000 people in the population.

### Measures

The variables analyzed in the study are age, an ordinal variable divided into three age groups (7–9, 10–14, 15–17, as we could not perform a continuous analysis), sector (Jewish, Arab), gender (male, female), and drowning sites (designated for bathing, non-designated for bathing), which are nominal variables. Drowning outcome (non-fatal drowning, fatal drowning) is a dichotomous variable.

The study population was divided into three age groups: 7–9 years, 10–14 years, and the older group of 15–17 years. This division was intended to compare the drowning rate to those age groups reported in the literature, thereby comparing Israel's drowning rates to other countries.

The age groups in the current study are partially similar to the age groups in the literature (the age range is usually between 0 years and older), while in the current study, the age range is 7–17. Therefore, a division was made that would be similar or as close as possible to the ages proposed in the literature.

The reference group in this study is the age group of 7–9, the youngest group participating in the analysis used for comparison with the other groups (10–14, 15–17).

The study examines the factors that may explain disparities between different groups divided by age, gender, sector (Jewish versus non-Jewish population consisting of Western Muslims and Christians, Druze, and Circassians), and drowning sites (designated sites intended for swimming versus natural disasters). In addition, examining these age groups may further help in understanding the gaps between the different age groups and drowning outcomes in the context of swimming lessons taught in Israel (ages 7–17) within the framework of schools under the supervision of the Ministry of Education.

### Statistical analysis

For the purpose of analyzing drowning rates, we used demographic data from the Central Bureau of Statistics for each age, divided by nationality per year. We made an annual average from the sample and divided it by the population. Next, we divided by 100,000 and thus obtained the drowning rate per 100,000.

The Z test for the distribution of proportions of continuous data (using a statistical significance of α = 0.05) was performed to examine the frequency of drowning in the Jewish and non-Jewish populations among males and females at different drowning sites (Tables 2, 3, 4, 5) to estimate the size of the population at risk of drowning (the frequency ratio in one group compared to another was tested, equal or different than 1, comparison between normalized frequencies per 100,000). The obtained value can be compared to the incidence rates reported for other countries in the world, thus allowing us to assess whether drowning rates in Israel are higher or lower than in other countries. For categorical data, we used the chi-square test.

In addition, the drownings were analyzed based on the drowning location, i.e., designated bathing sites (seas, pools, and lakes), places not designated for bathing (reservoirs, wells, cisterns, and oxidation pools), and natural disasters (rivers and floods). All results were displayed per 100,000 people.

The data were analyzed using the Excel software and R-statistics version.

## Results

### Demographic analysis of drowning data and drowning outcome

Table 1 presents data on 474 drowning cases in the period 2008-2018. The demographic characteristics of the drowning events in absolute numbers and the percentage of drownings, segmented by age groups (7-9,10-14,15-17), sector (Jewish, Arabs), the drowning outcome, and the site where the drowning occurred (designated bathing site, non-designated site for bathing, and natural disasters) are presented.

**Table 1:** Demographic characteristics and statistical analysis of drowning cases in 2008–2018 in Israel

Category	Subgroup	Designated for bathing	Non-designated for bathing	Disaster	*p*-value
Total cases	474	374 (78.9%)	26 (5.5%)	74 (15.6%)	–-
Age groups	age 7–9	72 (19.3%)	0 (0%)	12 (16.2%)	< 0.0001
	age 10–14	148 (39.6%)	2 (7.7%)	10 (13.5%)	
	age 15–17	154 (41.2%)	24 (92.3%)	52 (70.3%)	
Gender	Female	110 (29.4%)	4 (15.4%)	26 (35.1%)	0.1637
	Male	264 (70.6%)	22 (84.6%)	48 (64.9%)	
Sector	Jewish	283 (75.7%)	10 (38.5%)	62 (83.8%)	< 0.0001
	Arabs	91 (24.3%)	16 (61.5%)	12 (16.2%)	
Drowning outcome	Non-fatal	292 (78.1%)	19 (73.1%)	58 (78.4%)	0.8326
	Fatal	82 (21.9%)	7 (26.9%)	16 (21.6%)	

As observed in Table 1, the demographic data presented show that during the years 2008–2018, there were 474 drowning cases. There were 105 fatal cases (22.1) and 369 non-fatal drownings (77.8) among the population of children and adolescents in the age range of 7–17 years throughout the State of Israel. The cases occurred at sites intended for bathing, not intended for bathing, and as a result of natural disasters. The drowning cases were analyzed based on the place where the drowning occurred, that is: 374 (78.9) occurred in designated bathing sites (seas, pools, and lakes), 26 cases (5.5%) occurred in places not designated for bathing (reservoirs, wells, cisterns, and oxidation pools) and 74 cases (15.6%) were a result of disasters (rivers and floods).

The majority of drowning cases occurred in places designated for bathing and among the age group of 10 and older, a finding that was found to be significantly higher (*p* < 0.0001) than the other age groups. No significant difference was found between genders (*p* = 0.1637), but when comparing the Jewish and Arab populations, the rate of drowning among the Jewish population at sites designated for bathing and in disasters was significantly higher (more than 75% of the cases) than that of the Arab sector. Drowning among Arabs occurred primarily at sites not designated for bathing (more than 60% of the cases), a significantly higher value (*p* < 0.0001) than the other cases. Comparing the cases of fatal to non-fatal drowning, no difference was found between the various sites (designated for bathing, non-designated, and disasters) (*p* = 0.8326).

An analysis of the percentage of drownings (fatal and non-fatal) segmented by age subgroups showed that the oldest subgroup (15–17) accounted for 48% of all drowning cases, a statistically significantly high finding. In places not intended for bathing or in disaster scenarios, drowning among this subgroup (15–17) accounted for over 90%, while in all sites (designated, non-designated, and disaster sites), drowning in this age group amounted to 70% of all accidents The drowning, a difference that turned out to be statistically significantly higher.

The average rate of drownings is approximately 43 people per year, of which ~ 80% is non-fatal. In 2008, the survival rate was 72; in 2012, the rate was 62%, while in 2018, only 61% survived the drowning incident.

### Analysis of drowning rates based on age group

As indicated in Table 1, the drowning rate steadily increased with age. To estimate the extent of the increase in drowning risk, we set the young age group (7–9) as a reference and compared each age group to it, as presented in Table 2.

**Table 2:** Analysis of drowning rates by age group in Israel. Incidence rate per year, population per year (in thousands), and total drownings per 100,000 people

Drowning rates divided by groups, estimated per 100,000 children					
Age group	Incidence rate per year	Population per year	Total drownings	IRR	*p*-value
7–9	7.64	436.31	1.8	ref	
10–14	14.55	678.37	2.1	1.23	0.6496
15–17	20.91	378.82	5.5	3.15	0.0066

Table 2 compares the drowning rate per 100,000 between the following three age groups: 7–9, 10–14, and 15–17. Given that the reference group is the youngest age group, we found that the drowning rate in the 14–10 age group was 2.1/100,000, a rate that is 1.23 times higher than the reference group but without a significantly higher difference (*p* = 0.6496). However, in the adult age group, the drowning rate was 5.5 per 100,000, 3.15 times higher than in the youngest group, a significantly higher ratio (*p* = 0.0066).

### Analysis of the drowning data and drowning outcome based on gender

Table 3 compares the yearly average rate of drownings per 100,000 people between males and females with stratification by age group (7–9, 10–14, 15–17), sector (Jewish, Arabs), and drowning site (designated for bathing, non-designated for bathing, and disasters).

**Table 3:** Comparison and analysis of drowning rates based on gender with stratification by age groups, sector, drowning sites in Israel, and drowning outcome. The table refers to total drownings per 100,000 people, male (population 764.8 thousand), and female (population 728.7 thousand)

Category	Subgroup	Total drownings	Male	Female	IRR	*p*-value
***Total***	***––***	***2.89***	***3.97***	***1.75***	2.27	0.0139
Age groups	7–9	1.75	2.28	1.20	1.90	0.4024
	10–14	2.14	2.90	1.35	2.16	0.1769
	15–17	5.52	7.84	3.09	2.54	0.0576
Sector	Jewish	3.01	3.99	1.98	2.01	0.0641
	Arabs	2.56	3.92	1.14	3.43	0.0940
Drowning site	Designated for bathing	2.28	3.14	1.37	2.29	0.0280
	Non-designated for bathing	0.16	0.26	0.05	5.24	0.3582
	Disaster	0.45	0.57	0.32	1.76	0.4843
Drowning outcome	Non-fatal	2.25	3.05	1.40	2.17	0.0372
	Fatal	0.64	0.92	0.35	2.62	0.1881

As shown in Table 3, the drowning rate in males and females together amounts to 2.89/100,000. Gender comparison demonstrated a rate of drowning among males of 3.97/100,000, while in females, it reached 1.75/100,000, i.e., boys tend to drown statistically 2.27 times more (3.97/1.75) compared to girls. This difference is significantly higher (*p* = 0.0139).

In stratification by the different age groups, no significantly higher difference was found between males and females. Similarly, no statistically significant difference was found between the Jewish and Arab populations. At designated bathing sites, the rate of male drowning was 2.29 times higher (3.14/1.37) than females—a significantly higher difference (*p* = 0.0280). The number of drownings that did not end in death among males was 3.05/100,000 and among females 1.40/100,000, i.e., 2.17 (3.05/1.40) higher among males, a significantly higher difference (*p* = 0.0372). Death resulting from drowning in the general population stood at 0.64/100,000 cases per year. In males, the rate was 0.92/100,000, and in females 0.35/100,000 cases, with no significantly higher difference between the groups (*p* = 0.1881).

### Analysis of drowning data and outcome based on sector

Table 4 compares the yearly average of drowning cases per 100,000 people between the Jewish and Arab populations with stratification by age groups (7–9, 10–14,15–17), gender (male, female), and drowning site (designated for bathing, non-designated for bathing, and disasters).

**Table 4:** Comparison and analysis of drowning rates based on sector (Jewish and non-Jewish) with stratification by age groups, gender, drowning sites, and drowning outcome. The table refers to total drownings per 100,000 people, Arab population (population 421.9 thousand), and Jewish population (population 1071.6 thousand)

Category	Subgroup	Total drownings	Arabs	Jewish	IRR	*p*-value
***Total***	***––***	***2.89***	***2.56***	***3.01***	***0.85***	0.6467
Age groups	age 7–9	1.75	1.90	1.69	1.12	0.8843
	age 10–14	2.14	1.82	2.27	0.80	0.7173
	age 15–17	5.52	4.64	5.87	0.79	0.6458
Gender	Female	1.75	1.14	1.98	0.58	0.4452
	Male	3.97	3.92	3.99	0.98	0.9680
Drowning place	Designated for bathing	2.28	1.96	2.40	0.82	0.6122
	Non-designated for bathing	0.16	0.34	0.08	4.06	0.2943
	Disaster	0.45	0.26	0.53	0.49	0.4971
Drowning outcome	Non-fatal	2.25	1.72	2.45	0.70	0.4003
	Fatal	0.64	0.84	0.56	1.50	0.5446

As shown in Table 4, no significantly higher differences were found between the populations in all age groups, genders, drowning sites, and drowning outcomes. The drowning rates exhibited in the table show that the overall yearly drowning rate among the Jewish population was 3.01/100,000, and among the Arab population was 2.56/100,000, with no significantly higher differences (*p* = 0.6467). Regarding the drowning location, the drowning rate among the Jewish population was 2.40/100,000, while in the Arab population, the rate was 1.96/100,000, with no significantly higher difference (*p* = 0.6122). The drowning rate at sites not designated for bathing in the Jewish population was 0.08/100,000, while in the Arab population, it reached 0.34/100,000, a difference that is not significantly higher (*p* = 0.2943). The disaster rate in the Jewish population was 0.26/100,000, and in the Arab population 0.53/100,000, a difference that is not significantly higher (*p* = 0.4971). As for the drowning outcome, the rate of drownings that did not end in death among the Jewish population and others was 0.56/100,000, and in the Arab population, the rate was 0.84/100,000, a difference that is not significantly higher (*p* = 0.5546). The rate of drownings that ended in death among the Jewish population was 2.45/100,000 and 1.72/100,000 in the Arab sector, a difference that is not significantly higher (*p* = 0.4003).

### Analysis of drowning data and outcome based on swimming sites

Table 5 compares the yearly average of drowning cases per 100,000 people between sites designated for bathing and sites not designated for bathing, with stratification by age groups (7–9, 10–14, 15–17), gender, and sector.

**Table 5:** Comparison and analysis of drowning rates based on designated and non-designated bathing sites with stratification by age, gender, sector, and drowning outcome. The table refers to total drownings per 100,000 people, sites designated for bathing (population 1493.5 thousand), and non-designated for bathing (population 1493.5 thousand)

Category	Subgroup	Total drownings	Designated for bathing	Non-designated for bathing	IRR	*p*-value
***Total***	***––-***	***2.89***	***2.28***	***0.16***	14.38	0.0001
Age groups	7–9	1.75	1.50	0.00	–-	–-
	10–14	2.14	1.98	0.03	74.00	0.0683
	15–17	5.52	3.70	0.58	6.42	0.0107
Gender	Female	1.75	1.37	0.05	27.50	0.0496
	Male	3.97	3.14	0.26	12.00	0.0007
Sector	Jewish	3.01	2.40	0.08	28.30	0.0017
	Arab	2.56	1.96	0.34	5.69	0.0532
Drowning outcome	Non-fatal	2.25	1.78	0.12	15.37	0.0005
	Fatal	0.64	0.50	0.04	11.71	0.0595

As revealed in Table 5, the drowning rate at sites designated for bathing, which amounts to 14.38 (2.28/0.16), is higher compared with sites not designated for bathing, a significantly higher result (*p* < 0.0001).

Drowning rates in the various age groups (fatal and non-fatal drowning) demonstrate that in the age group of 7–9, the drowning rate at sites designated for bathing was 1.5/100,000, whereas, at non-designated sites, the rate was 0.00/100,000. In the age group of 10–14, the drowning rate in sites designated for bathing was 1.98/100,000 and 0.03/100,000 in non-designated sites – a difference that is not significantly higher (*p* = 0.0683). In the age group of 15–17, the rate of drowning in sites designated for bathing was 3.70/100,000 and 0.58/100,000 in non-designated sites – a difference that was found to be significantly higher (*p* = 0.0107). The age group of 15–17 exhibited an increased risk of drowning 6.42 times (3.70/0.58) higher at designated sites compared with sites that are not intended for bathing (*p* = 0.0107). Girls were 27.50 times (1.37/0.05) more prone to drowning in sites designated for bathing compared with non-designated sites, which is a significantly higher finding (*p* = 0.0496). For boys, the risk of drowning at sites designated for bathing was 12.00 times higher (0.12/3.14) at sites not designated for bathing, which is a significantly higher finding (*p* = 0.0007).

Stratification by sectors revealed that in the Jewish population, the risk of drowning at sites designated for bathing was 28.30 times higher than at non-designated sites (2.40/0.08), a significantly higher finding (*p* = 0.0017). On the other hand, among the Arab population, no significantly higher difference was found between the rate of drowning at designated and non-designated sites (*p* = 0.0532).

Non-fatal drowning cases were 15.37 times higher (1.78/0.12) in non-designated sites than in non-bathing sites, a significantly higher finding (*p* = 0.0005).

Drowning death at designated bathing sites was found to be at a rate of 0.50/100,000, 11.71 times higher compared with sites not designated for bathing, where the rate is 0.04/100,000. These findings are not significantly higher (*p* = 0.0595).

A comparative analysis between designated sites, non-designated sites, and disasters was not performed as disasters are sporadic, unplanned events, as opposed to proactive swimming.

## Discussion

In Israel, about 40 cases of drowning occur on average per year among children and adolescents in the age range of 7–17, of which about eight cases end in death. In this pioneering study, we examined the rate of drowning in Israel for this age group while stratifying by three subgroups (7–9, 10–14, 15–17), i.e., gender, sector, drowning site, and drowning outcome.

Out of 474 drowning cases during 2008–2018, 22% had a fatal outcome. Most of the drowning cases (374), which constitute 78.9% of all cases, occurred in water sources designated for bathing. Most drownings (59.1%) occurred among boys, at a rate approximately 1.5 higher than girls (a statistically insignificant result). These findings are similar to the results published in [4] and [3].

The drowning rates compared to what is reported in the literature show that the non-fatal drowning rate in the 7–17 age group was 2.25/100,000, similar to the findings of an American study that examined the rate of fatal drownings among children and teenagers in the 5–19 age range (a more comprehensive range) during the years 1993–2008. The results range from 0.8 to 2.6 cases per 100,000 people [10]. Furthermore, the fatal drowning rate in our study was 0.64/100,000, lower than the rate of 0.81/100,000 found in the U.S. for a narrower age subgroup (5–14) during 2001–2002 [11]. However, our results correspond to the range of 0.6/100,000 to 0.7/100,000 found in Norway, Poland, Japan, and South Korea [12].

The ratio between non-fatal and fatal drownings in Israel was 3.51 (2.25/0.64), a value commensurate with the range of 2 to 4 reported by an Australian study that examined non-fatal relative to fatal drowning rates between 2002 and 2015.

A further finding of our study was that the relative ratio between non-fatal drowning and drowning in a site designated for bathing was 3.56 (1.78/0.5), similar to the aforementioned Australian study that found a ratio of 4.35 [13].

The non-fatal drowning rate among males in sites designated for bathing (3.14/100,000) was 2.17 times higher than among females (1.37/100,000), a significantly higher result (*p* = 0.03), similar to data from other countries [3, 7].

Non-fatal drowning outcome was also higher at sites designated for bathing compared with non-designated sites. These findings are lower than those reported [7] for designated sites (7.9/100,000 in natural water sources and 18.8/100,000 in swimming pools).

Comparing the Jewish majority to the Arab-Muslim minority, no significant difference was found in drowning rates based on stratification by age, gender, designated/non-designated sites for bathing or disasters, and fatal and non-fatal drownings. However, the Arab minority drowned in larger percentages (more than 60% of all cases) in places not designated for bathing. These findings differ from that reported by [7], which indicates a higher drowning rate among Hispanics and African Americans compared to the Caucasian population in the United States [7].

The highest non-fatal drowning rates occurred in places designated for bathing, especially among the Jewish older youth subgroup (ages 15–17).

We found that among the oldest Jewish subgroup (15–17), the drowning rate at designated bathing sites was 6.42 times higher than at non-bathing sites (*p* = 0.0107). In this exact age group, one of the most severe risk factors for drowning is alcohol consumption before and during bathing [6, 14]. Although we did not examine whether the drowning individual consumed alcohol before the incident, we note that for the Arab-Muslim population in Israel, the consumption of alcohol is prohibited according to religion, which may explain the difference observed in the drowning rates between Jewish and Arab populations (we did not take into account the Christian Arab population, since it constitutes only 7% of the entire Israeli Arab population). Moreover, in our study, we found that the rate of drowning increases with age, contrary to the results published in an American study [7]. Therefore, it is important to raise awareness among youth of the dangers associated with swimming in sites not designated for bathing or in designated sites without lifeguard supervision and to educate the youth about the dangers associated with alcohol consumption before bathing and reckless behavior in the water. Designated bathing places with lifeguards and supervision services should provide a safe environment that minimizes drowning incidents.

However, we found that the highest number of drowning incidents occurred in places designated for bathing. It is possible that the high drowning rates may be due to increased exposure. Therefore, this value is alarming and requires examining the reasons leading to drowning in places that are supposed to be safe. Hence, there is room for a thorough examination and re-evaluation of the instructions for the supervision of bathers at the various bathing sites.

## Limitations

Certain data are missing in the hospital databases. The Jewish population constitutes 73.9% of the general population in Israel, while the Arab population (Muslims, Christians, Druze, and Circassians) amounts to 21.1%. The non-Jewish, non-Arab population (estimated to be 5% of the general population) was not recorded separately from the Jewish population and therefore considered in the same group as the Jewish population. Comparison of drowning rates between Israel and other countries worldwide refers in part to identical age groups, especially between the ages of 10–14 and 15–17. No data on drowning rates of other age groups were found for comparison.

## Conclusion and practical applications

On average, approximately 40 avoidable drownings occur per year in Israel in the 7–17 age group. Education and information are essential tools for raising awareness and reducing the number of drowning incidents among children and adolescents.

There is a need to continue research in this field to better understand the circumstances leading children and teenagers to drown in designated bathing areas and the specific circumstances of the context surrounding drowning cases in Israel.

Also, due to the high number of drownings in places designated for bathing, we recommend putting emphasis on explaining the importance of entering the water only at designated bathing sites with the presence of a lifeguard and avoiding taking unnecessary risks.

We recommend that the decision-makers re-examine the guidelines and measures taken to maintain safety in sites designated for bathing.

In addition, it is necessary to continue investigating whether alcohol consumption and risky behavior typical of 15–17 year-olds are related to the drowning cases in the current study.

## Data Availability

The data that support the findings of this study are available from Beterem organization, but restrictions apply to the availability of these data, which were used under license for the current study, and so are not publicly available. Data are, however, available from the authors upon reasonable request and with permission of Beterem organization. Please contact Sigalit Abihasira at sigal00000@gmail.com; Department of Health Systems Management, Faculty of Health Sciences, Ariel University, Ariel 40,700, Israel.
